# 1,1,3,3,5,5,7,7-Octa­phenyl-2,6-dioxa-4,8-diaza-1,3,5,7-tetra­silacyclo­octa­ne

**DOI:** 10.1107/S1600536811014851

**Published:** 2011-04-29

**Authors:** Zhen Lv, Lina Dai, Xuezhong Zhang, Zhijie Zhang, Zemin Xie

**Affiliations:** a Institute of Chemistry, Chinese Academy of Sciences, Beijing 100190, People’s Republic of China

## Abstract

The title mol­ecule, C_48_H_42_N_2_O_2_Si_4_, lies on a twofold rotation axis. The eight-membered ring has a slightly distorted boat conformation.

## Related literature

For the hydrolysis of 1,3-bis-(hy­droxy­diphenyl­silan­yl)-2,2,4,4-tetra­phenyl­cyclo­disilazane, see: Voronkov *et al.* (1977 ▶).
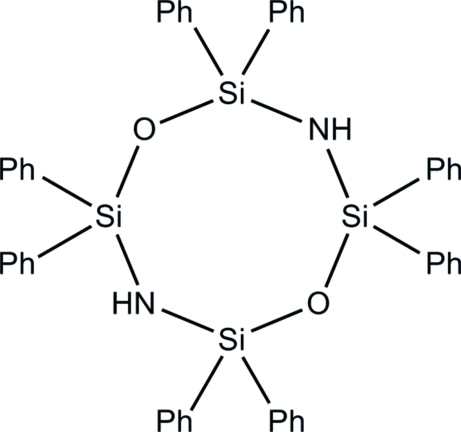


## Experimental

### 

#### Crystal data

C_48_H_42_N_2_O_2_Si_4_
*M* *_r_* = 791.20
Monoclinic, 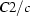

*a* = 12.1188 (18) Å
*b* = 17.016 (3) Å
*c* = 20.621 (3) Å
β = 93.216 (3)°
*V* = 4245.8 (11) Å^3^
*Z* = 4
Mo *K*α radiation
μ = 0.18 mm^−1^
*T* = 173 K
0.35 × 0.35 × 0.05 mm

#### Data collection

Rigaku MM007-HF CCD (Saturn 724+) diffractometer
Absorption correction: multi-scan (*CrystalClear*; Rigaku, 2007 ▶) *T*_min_ = 0.939, *T*_max_ = 0.991
14029 measured reflections
4833 independent reflections
4411 reflections with *I* > 2σ(*I*)
*R*_int_ = 0.039

#### Refinement

*R*[*F* ^2^ > 2σ(*F* ^2^)] = 0.052
*wR*(*F* ^2^) = 0.120
*S* = 1.10
4833 reflections
253 parameters
H-atom parameters constrained
Δρ_max_ = 0.39 e Å^−3^
Δρ_min_ = −0.30 e Å^−3^

### 

Data collection: *CrystalClear* (Rigaku, 2007 ▶); cell refinement: *CrystalClear*; data reduction: *CrystalClear*; program(s) used to solve structure: *SHELXS97* (Sheldrick, 2008 ▶); program(s) used to refine structure: *SHELXL97* (Sheldrick, 2008 ▶); molecular graphics: *SHELXTL* (Sheldrick, 2008 ▶); software used to prepare material for publication: *SHELXL97*.

## Supplementary Material

Crystal structure: contains datablocks I, global. DOI: 10.1107/S1600536811014851/lh5233sup1.cif

Structure factors: contains datablocks I. DOI: 10.1107/S1600536811014851/lh5233Isup2.hkl

Supplementary material file. DOI: 10.1107/S1600536811014851/lh5233Isup3.cml

Additional supplementary materials:  crystallographic information; 3D view; checkCIF report

## Figures and Tables

**Fig. 1.**
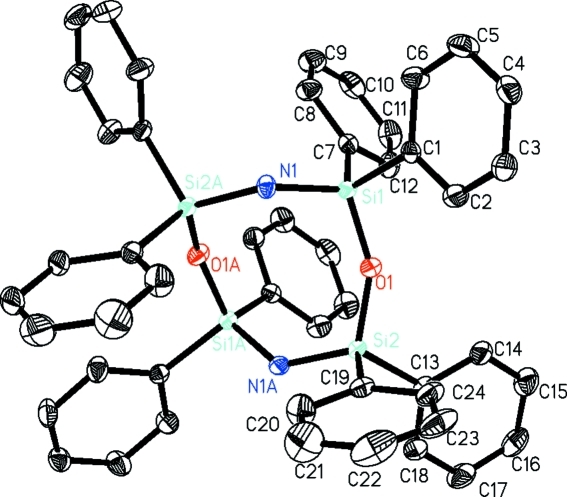
The molecular structure of the title compound with 30% ellipsoids. H atoms are not shown (symmetry code (A): -x+1, y, -z+3/2).

**Fig. 2.**
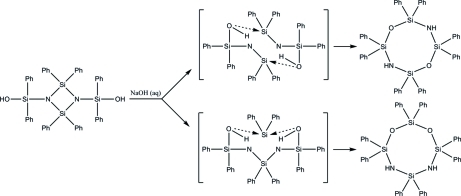
The hydrolysis reaction of 1,3-bis-(hydroxydiphenylsilanyl)-2,2,4,4-tetraphenylcyclodisilazane

## supplementary crystallographic information

### Comment

The title compound is one of the hydrolysis products of 1,3-bis-(hydroxydiphenylsilanyl)-2,2,4,4-tetraphenylcyclodisilazane. Voronkov *et al.* (1977) reported that the hydrolysis of 1,3-bis-(hydroxydiphenylsilanyl)-2,2,4,4-tetraphenylcyclodisilazane in base medium could give 1,1,3,3,5,5,7,7-octaphenyl-2,4-dioxa-6,8,-diaza-1,3,5,7-tetrasilacyclo-octane and we have found that the title compound was also produced. Its crystal structure is presented herein. The molecular structure of the title compound is shown in Fig. 1. The eight-membered ring has a slightly disorted boat conformation.

### Experimental

The reaction scheme is shown in Fig. 2. 1 ml aqueous solution of sodium hydroxide (0.1 mol/*L*) was added to a solution of 1,3-bis-(hydroxydiphenylsilanyl)-2,2,4,4-tetraphenylcyclodisilazane (1 g) in tetrahydrofuran (10 ml). After stirring for 30 min at room temprature, the solvents were removed under reduced pressure. The crude product was recrystallized from n-hexane to give colorless crystals.

### Refinement

All the H atoms were located in difference maps but were subsequently placed in calculated positions with C-H = 0.95Å and constrained in a riding-model approximation with *U*_iso_(H) = 1.2*U*_eq_(C).

### Crystal data

**Table:**

C_48_H_42_N_2_O_2_Si_4_	*F*(000) = 1664
*M**_r_* = 791.20	*D*_x_ = 1.238 Mg m^−^^3^
Monoclinic, *C*2/*c*	Mo *K*α radiation, λ = 0.71073 Å
Hall symbol: -C 2yc	Cell parameters from 7324 reflections
*a* = 12.1188 (18) Å	θ = 2.1–27.5°
*b* = 17.016 (3) Å	µ = 0.18 mm^−^^1^
*c* = 20.621 (3) Å	*T* = 173 K
β = 93.216 (3)°	Platelet, colorless
*V* = 4245.8 (11) Å^3^	0.35 × 0.35 × 0.05 mm
*Z* = 4	

### Data collection

**Table:**

Rigaku MM007-HF CCD (Saturn 724+) diffractometer	4833 independent reflections
Radiation source: rotating anode	4411 reflections with *I* > 2σ(*I*)
Confocal	*R*_int_ = 0.039
ω scans at fixed χ = 45°	θ_max_ = 27.5°, θ_min_ = 2.1°
Absorption correction: multi-scan (*CrystalClear*; Rigaku, 2007)	*h* = −15→13
*T*_min_ = 0.939, *T*_max_ = 0.991	*k* = −18→22
14029 measured reflections	*l* = −26→24

### Refinement

**Table:**

Refinement on *F*^2^	Primary atom site location: structure-invariant direct methods
Least-squares matrix: full	Secondary atom site location: difference Fourier map
*R*[*F*^2^ > 2σ(*F*^2^)] = 0.052	Hydrogen site location: inferred from neighbouring sites
*wR*(*F*^2^) = 0.120	H-atom parameters constrained
*S* = 1.10	*w* = 1/[σ^2^(*F*_o_^2^) + (0.0442*P*)^2^ + 4.974*P*] where *P* = (*F*_o_^2^ + 2*F*_c_^2^)/3
4833 reflections	(Δ/σ)_max_ = 0.001
253 parameters	Δρ_max_ = 0.39 e Å^−^^3^
0 restraints	Δρ_min_ = −0.30 e Å^−^^3^

### Special details

**Table:**

Geometry. All e.s.d.'s (except the e.s.d. in the dihedral angle between two l.s. planes) are estimated using the full covariance matrix. The cell e.s.d.'s are taken into account individually in the estimation of e.s.d.'s in distances, angles and torsion angles; correlations between e.s.d.'s in cell parameters are only used when they are defined by crystal symmetry. An approximate (isotropic) treatment of cell e.s.d.'s is used for estimating e.s.d.'s involving l.s. planes.
Refinement. Refinement of *F*^2^ against ALL reflections. The weighted *R*-factor *wR* and goodness of fit *S* are based on *F*^2^, conventional *R*-factors *R* are based on *F*, with *F* set to zero for negative *F*^2^. The threshold expression of *F*^2^ > σ(*F*^2^) is used only for calculating *R*-factors(gt) *etc*. and is not relevant to the choice of reflections for refinement. *R*-factors based on *F*^2^ are statistically about twice as large as those based on *F*, and *R*- factors based on ALL data will be even larger.

### Fractional atomic coordinates and isotropic or equivalent isotropic displacement parameters (Å^2^)

**Table:**

	*x*	*y*	*z*	*U*_iso_*/*U*_eq_	
Si1	0.34094 (4)	0.15720 (3)	0.71343 (2)	0.02431 (13)	
Si2	0.55767 (4)	0.21644 (3)	0.65217 (2)	0.02461 (13)	
O1	0.44552 (11)	0.16871 (8)	0.66768 (6)	0.0324 (3)	
N1	0.35191 (13)	0.21530 (9)	0.78113 (7)	0.0282 (3)	
H1A	0.3008	0.2520	0.7818	0.034*	
C1	0.21399 (15)	0.18949 (11)	0.66561 (8)	0.0269 (4)	
C2	0.21930 (18)	0.23932 (15)	0.61226 (10)	0.0437 (5)	
H2A	0.2895	0.2534	0.5975	0.052*	
C3	0.12476 (19)	0.26890 (15)	0.58017 (11)	0.0471 (6)	
H3A	0.1310	0.3036	0.5445	0.057*	
C4	0.02258 (18)	0.24847 (14)	0.59954 (10)	0.0415 (5)	
H4A	−0.0422	0.2684	0.5772	0.050*	
C5	0.01439 (18)	0.19908 (15)	0.65148 (12)	0.0478 (6)	
H5A	−0.0564	0.1845	0.6651	0.057*	
C6	0.10912 (17)	0.17011 (13)	0.68447 (11)	0.0390 (5)	
H6A	0.1019	0.1363	0.7207	0.047*	
C7	0.33633 (15)	0.05141 (11)	0.73523 (9)	0.0284 (4)	
C8	0.29044 (18)	0.02462 (12)	0.79170 (11)	0.0405 (5)	
H8A	0.2599	0.0613	0.8204	0.049*	
C9	0.2889 (2)	−0.05507 (14)	0.80646 (12)	0.0503 (6)	
H9A	0.2573	−0.0725	0.8450	0.060*	
C10	0.3333 (2)	−0.10885 (13)	0.76511 (13)	0.0492 (6)	
H10A	0.3335	−0.1632	0.7756	0.059*	
C11	0.3772 (2)	−0.08378 (13)	0.70882 (12)	0.0481 (6)	
H11A	0.4062	−0.1209	0.6799	0.058*	
C12	0.37953 (19)	−0.00461 (12)	0.69411 (10)	0.0390 (5)	
H12A	0.4111	0.0121	0.6553	0.047*	
C13	0.61490 (16)	0.16531 (11)	0.58150 (8)	0.0289 (4)	
C14	0.5520 (2)	0.11582 (13)	0.54049 (10)	0.0416 (5)	
H14A	0.4766	0.1068	0.5485	0.050*	
C15	0.5975 (2)	0.07921 (15)	0.48789 (12)	0.0550 (6)	
H15A	0.5531	0.0461	0.4600	0.066*	
C16	0.7078 (2)	0.09105 (15)	0.47616 (12)	0.0545 (7)	
H16A	0.7399	0.0647	0.4412	0.065*	
C17	0.7700 (2)	0.14071 (17)	0.51506 (12)	0.0543 (6)	
H17A	0.8451	0.1501	0.5065	0.065*	
C18	0.72369 (18)	0.17780 (15)	0.56732 (11)	0.0434 (5)	
H18A	0.7679	0.2125	0.5938	0.052*	
C19	0.52733 (15)	0.32134 (11)	0.63098 (9)	0.0306 (4)	
C20	0.5545 (2)	0.38324 (14)	0.67257 (12)	0.0515 (6)	
H20A	0.5909	0.3725	0.7136	0.062*	
C21	0.5299 (3)	0.46049 (16)	0.65572 (16)	0.0752 (9)	
H21A	0.5515	0.5019	0.6846	0.090*	
C22	0.4745 (3)	0.47717 (17)	0.59751 (16)	0.0731 (9)	
H22A	0.4558	0.5299	0.5866	0.088*	
C23	0.4461 (2)	0.41749 (17)	0.55499 (13)	0.0591 (7)	
H23A	0.4083	0.4290	0.5145	0.071*	
C24	0.47270 (17)	0.34019 (14)	0.57119 (10)	0.0410 (5)	
H24A	0.4535	0.2994	0.5412	0.049*	

### Atomic displacement parameters (Å^2^)

**Table:**

	*U*^11^	*U*^22^	*U*^33^	*U*^12^	*U*^13^	*U*^23^
Si1	0.0250 (2)	0.0252 (2)	0.0230 (2)	−0.00224 (18)	0.00372 (18)	−0.00254 (18)
Si2	0.0258 (3)	0.0269 (2)	0.0213 (2)	−0.00168 (19)	0.00308 (18)	0.00178 (18)
O1	0.0313 (7)	0.0380 (7)	0.0284 (6)	−0.0055 (6)	0.0064 (5)	−0.0020 (6)
N1	0.0297 (8)	0.0287 (8)	0.0260 (7)	0.0041 (6)	0.0004 (6)	−0.0057 (6)
C1	0.0293 (9)	0.0260 (8)	0.0254 (8)	−0.0021 (7)	0.0009 (7)	−0.0058 (7)
C2	0.0334 (11)	0.0624 (14)	0.0353 (11)	−0.0068 (10)	0.0012 (9)	0.0122 (10)
C3	0.0423 (12)	0.0627 (15)	0.0356 (11)	−0.0032 (11)	−0.0053 (9)	0.0159 (10)
C4	0.0353 (11)	0.0478 (12)	0.0404 (11)	0.0012 (9)	−0.0074 (9)	−0.0009 (10)
C5	0.0268 (10)	0.0570 (14)	0.0595 (14)	−0.0025 (10)	0.0014 (10)	0.0140 (12)
C6	0.0333 (10)	0.0390 (11)	0.0449 (12)	−0.0033 (9)	0.0038 (9)	0.0096 (9)
C7	0.0272 (9)	0.0271 (9)	0.0307 (9)	−0.0005 (7)	−0.0005 (7)	−0.0023 (7)
C8	0.0443 (12)	0.0326 (10)	0.0455 (12)	−0.0048 (9)	0.0114 (9)	0.0010 (9)
C9	0.0555 (15)	0.0413 (12)	0.0544 (14)	−0.0116 (11)	0.0062 (11)	0.0125 (11)
C10	0.0524 (14)	0.0262 (10)	0.0674 (16)	−0.0051 (9)	−0.0129 (12)	0.0027 (10)
C11	0.0611 (15)	0.0301 (11)	0.0521 (13)	0.0066 (10)	−0.0070 (11)	−0.0091 (10)
C12	0.0493 (12)	0.0333 (10)	0.0340 (10)	0.0054 (9)	−0.0003 (9)	−0.0042 (8)
C13	0.0335 (10)	0.0305 (9)	0.0232 (8)	0.0009 (7)	0.0058 (7)	0.0036 (7)
C14	0.0483 (13)	0.0445 (12)	0.0328 (10)	−0.0049 (10)	0.0081 (9)	−0.0050 (9)
C15	0.0738 (18)	0.0509 (14)	0.0413 (12)	−0.0061 (13)	0.0116 (12)	−0.0146 (11)
C16	0.0748 (18)	0.0516 (14)	0.0393 (12)	0.0140 (13)	0.0235 (12)	−0.0043 (11)
C17	0.0447 (13)	0.0709 (17)	0.0494 (14)	0.0074 (12)	0.0219 (11)	0.0007 (12)
C18	0.0358 (11)	0.0563 (14)	0.0393 (11)	−0.0031 (10)	0.0110 (9)	−0.0057 (10)
C19	0.0295 (9)	0.0315 (9)	0.0315 (9)	0.0007 (7)	0.0073 (7)	0.0071 (8)
C20	0.0683 (17)	0.0350 (12)	0.0507 (13)	0.0018 (11)	−0.0019 (12)	−0.0010 (10)
C21	0.113 (3)	0.0323 (13)	0.081 (2)	0.0055 (15)	0.0110 (19)	−0.0008 (13)
C22	0.094 (2)	0.0425 (15)	0.086 (2)	0.0219 (15)	0.0350 (19)	0.0248 (15)
C23	0.0513 (14)	0.0715 (18)	0.0562 (15)	0.0179 (13)	0.0186 (12)	0.0386 (14)
C24	0.0368 (11)	0.0514 (13)	0.0355 (11)	0.0043 (9)	0.0075 (9)	0.0163 (9)

### Geometric parameters (Å, °)

**Table:**

Si1—O1	1.6338 (14)	C10—C11	1.372 (4)
Si1—N1	1.7099 (15)	C10—H10A	0.9500
Si1—C7	1.8571 (19)	C11—C12	1.381 (3)
Si1—C1	1.8630 (19)	C11—H11A	0.9500
Si2—O1	1.6302 (14)	C12—H12A	0.9500
Si2—N1^i^	1.7098 (15)	C13—C18	1.383 (3)
Si2—C13	1.8643 (19)	C13—C14	1.390 (3)
Si2—C19	1.869 (2)	C14—C15	1.392 (3)
N1—Si2^i^	1.7098 (15)	C14—H14A	0.9500
N1—H1A	0.8800	C15—C16	1.386 (4)
C1—C6	1.390 (3)	C15—H15A	0.9500
C1—C2	1.393 (3)	C16—C17	1.363 (4)
C2—C3	1.385 (3)	C16—H16A	0.9500
C2—H2A	0.9500	C17—C18	1.394 (3)
C3—C4	1.367 (3)	C17—H17A	0.9500
C3—H3A	0.9500	C18—H18A	0.9500
C4—C5	1.369 (3)	C19—C20	1.386 (3)
C4—H4A	0.9500	C19—C24	1.403 (3)
C5—C6	1.391 (3)	C20—C21	1.388 (3)
C5—H5A	0.9500	C20—H20A	0.9500
C6—H6A	0.9500	C21—C22	1.372 (4)
C7—C8	1.395 (3)	C21—H21A	0.9500
C7—C12	1.397 (3)	C22—C23	1.373 (4)
C8—C9	1.390 (3)	C22—H22A	0.9500
C8—H8A	0.9500	C23—C24	1.390 (3)
C9—C10	1.381 (4)	C23—H23A	0.9500
C9—H9A	0.9500	C24—H24A	0.9500
			
O1—Si1—N1	112.01 (8)	C11—C10—H10A	120.0
O1—Si1—C7	106.91 (8)	C9—C10—H10A	120.0
N1—Si1—C7	111.40 (8)	C10—C11—C12	120.2 (2)
O1—Si1—C1	107.65 (8)	C10—C11—H11A	119.9
N1—Si1—C1	106.60 (8)	C12—C11—H11A	119.9
C7—Si1—C1	112.27 (8)	C11—C12—C7	121.3 (2)
O1—Si2—N1^i^	109.90 (7)	C11—C12—H12A	119.4
O1—Si2—C13	105.76 (8)	C7—C12—H12A	119.4
N1^i^—Si2—C13	111.95 (8)	C18—C13—C14	117.47 (18)
O1—Si2—C19	111.49 (8)	C18—C13—Si2	119.63 (15)
N1^i^—Si2—C19	107.92 (8)	C14—C13—Si2	122.87 (15)
C13—Si2—C19	109.86 (8)	C13—C14—C15	121.1 (2)
Si2—O1—Si1	148.79 (9)	C13—C14—H14A	119.4
Si2^i^—N1—Si1	132.87 (10)	C15—C14—H14A	119.4
Si2^i^—N1—H1A	113.6	C16—C15—C14	120.0 (2)
Si1—N1—H1A	113.6	C16—C15—H15A	120.0
C6—C1—C2	116.67 (18)	C14—C15—H15A	120.0
C6—C1—Si1	121.53 (15)	C17—C16—C15	119.6 (2)
C2—C1—Si1	121.61 (15)	C17—C16—H16A	120.2
C3—C2—C1	121.7 (2)	C15—C16—H16A	120.2
C3—C2—H2A	119.2	C16—C17—C18	120.2 (2)
C1—C2—H2A	119.2	C16—C17—H17A	119.9
C4—C3—C2	120.4 (2)	C18—C17—H17A	119.9
C4—C3—H3A	119.8	C13—C18—C17	121.6 (2)
C2—C3—H3A	119.8	C13—C18—H18A	119.2
C3—C4—C5	119.4 (2)	C17—C18—H18A	119.2
C3—C4—H4A	120.3	C20—C19—C24	117.0 (2)
C5—C4—H4A	120.3	C20—C19—Si2	122.97 (16)
C4—C5—C6	120.4 (2)	C24—C19—Si2	120.04 (16)
C4—C5—H5A	119.8	C19—C20—C21	121.6 (2)
C6—C5—H5A	119.8	C19—C20—H20A	119.2
C1—C6—C5	121.4 (2)	C21—C20—H20A	119.2
C1—C6—H6A	119.3	C22—C21—C20	120.2 (3)
C5—C6—H6A	119.3	C22—C21—H21A	119.9
C8—C7—C12	117.66 (18)	C20—C21—H21A	119.9
C8—C7—Si1	122.53 (15)	C21—C22—C23	119.9 (2)
C12—C7—Si1	119.81 (15)	C21—C22—H22A	120.0
C9—C8—C7	120.8 (2)	C23—C22—H22A	120.0
C9—C8—H8A	119.6	C22—C23—C24	120.0 (2)
C7—C8—H8A	119.6	C22—C23—H23A	120.0
C10—C9—C8	120.1 (2)	C24—C23—H23A	120.0
C10—C9—H9A	120.0	C23—C24—C19	121.2 (2)
C8—C9—H9A	120.0	C23—C24—H24A	119.4
C11—C10—C9	120.0 (2)	C19—C24—H24A	119.4
			
N1^i^—Si2—O1—Si1	55.8 (2)	C9—C10—C11—C12	1.5 (4)
C13—Si2—O1—Si1	176.78 (17)	C10—C11—C12—C7	−0.9 (3)
C19—Si2—O1—Si1	−63.8 (2)	C8—C7—C12—C11	−0.2 (3)
N1—Si1—O1—Si2	−7.6 (2)	Si1—C7—C12—C11	−179.88 (17)
C7—Si1—O1—Si2	−129.94 (18)	O1—Si2—C13—C18	−162.77 (16)
C1—Si1—O1—Si2	109.24 (18)	N1^i^—Si2—C13—C18	−43.10 (19)
O1—Si1—N1—Si2^i^	−66.15 (15)	C19—Si2—C13—C18	76.79 (18)
C7—Si1—N1—Si2^i^	53.55 (15)	O1—Si2—C13—C14	18.92 (19)
C1—Si1—N1—Si2^i^	176.34 (12)	N1^i^—Si2—C13—C14	138.60 (17)
O1—Si1—C1—C6	164.56 (16)	C19—Si2—C13—C14	−101.52 (18)
N1—Si1—C1—C6	−75.08 (18)	C18—C13—C14—C15	1.1 (3)
C7—Si1—C1—C6	47.16 (18)	Si2—C13—C14—C15	179.47 (18)
O1—Si1—C1—C2	−20.62 (19)	C13—C14—C15—C16	0.8 (4)
N1—Si1—C1—C2	99.73 (18)	C14—C15—C16—C17	−2.2 (4)
C7—Si1—C1—C2	−138.02 (17)	C15—C16—C17—C18	1.6 (4)
C6—C1—C2—C3	0.8 (3)	C14—C13—C18—C17	−1.7 (3)
Si1—C1—C2—C3	−174.23 (19)	Si2—C13—C18—C17	179.86 (19)
C1—C2—C3—C4	−1.3 (4)	C16—C17—C18—C13	0.4 (4)
C2—C3—C4—C5	0.7 (4)	O1—Si2—C19—C20	107.10 (19)
C3—C4—C5—C6	0.2 (4)	N1^i^—Si2—C19—C20	−13.7 (2)
C2—C1—C6—C5	0.1 (3)	C13—Si2—C19—C20	−135.99 (19)
Si1—C1—C6—C5	175.18 (18)	O1—Si2—C19—C24	−71.58 (17)
C4—C5—C6—C1	−0.6 (4)	N1^i^—Si2—C19—C24	167.65 (15)
O1—Si1—C7—C8	153.74 (16)	C13—Si2—C19—C24	45.33 (18)
N1—Si1—C7—C8	31.06 (19)	C24—C19—C20—C21	−0.6 (4)
C1—Si1—C7—C8	−88.42 (18)	Si2—C19—C20—C21	−179.4 (2)
O1—Si1—C7—C12	−26.55 (18)	C19—C20—C21—C22	2.0 (5)
N1—Si1—C7—C12	−149.23 (15)	C20—C21—C22—C23	−1.9 (5)
C1—Si1—C7—C12	91.29 (17)	C21—C22—C23—C24	0.6 (4)
C12—C7—C8—C9	0.5 (3)	C22—C23—C24—C19	0.8 (4)
Si1—C7—C8—C9	−179.76 (18)	C20—C19—C24—C23	−0.7 (3)
C7—C8—C9—C10	0.1 (4)	Si2—C19—C24—C23	178.04 (17)
C8—C9—C10—C11	−1.2 (4)		

Symmetry codes: (i) −*x*+1, *y*, −*z*+3/2.
